# Preparation and Characterization of Chitosan/Polyvinyl Alcohol/Zinc Gluconate Hydrogel: Antibacterial and Zinc Ion Release

**DOI:** 10.3390/polym17233099

**Published:** 2025-11-21

**Authors:** Yujia Wang, Yanling Bao, Yongtao Yao, Sijia Chen, Wenpeng Tang, Jiawen Liu, Juncheng Wang, Zhigang Li, Bo Tian, Shibo Wu, Hongtao Zhao, Pengfei Huo, Jidong Dong, Dawei Zhang

**Affiliations:** 1 Laboratory of Woody Oil Resources Utilization, Northeast Forestry University, Harbin 150040, China; wangyujia990508@163.com (Y.W.); ciga0623@163.com (S.C.); xi78495@163.com (W.T.); 18870462042@163.com (J.L.); jcwang514@163.com (J.W.); s548_4@163.com (S.W.); huopengfei@nefu.edu.cn (P.H.); 2 Xi’an Aerospace Composites Research Institute, Xi’an 710025, China; m1779_flaktoczf3@aka.yeah.net; 3 National Key Laboratory of Science and Technology on Advanced Composites in Special Environments, Harbin Institute of Technology, Harbin 150000, China; yaoyt@hit.edu.cn; 4 Heilongjiang Institute of Atomic Energy, Harbin 150081, China; lzg0015@163.com (Z.L.); tianbo_0627@163.com (B.T.); zhaohongtao2019@163.com (H.Z.)

**Keywords:** zinc gluconate, antibacterial activity, cytotoxicity, gamma-ray irradiation, CS/PVA hydrogel

## Abstract

Zinc ions, as essential trace elements in the human body, play a crucial role in promoting wound healing. They have significant applications in the medical field. In this paper, chitosan (CS)/polyvinyl alcohol (PVA)/zinc gluconate hydrogel was synthesized via gamma ray irradiation cross-linking. The hydrogel exhibited excellent antibacterial properties, and could continuously release zinc ions. Antibacterial assays demonstrated that the combination of CS and zinc ions improved the antibacterial properties of hydrogel. The inhibition zones against both *Staphylococcus aureus* and *Escherichia coli* exceed 12 mm. The cell viability can reach 108.25%. The prepared hydrogel could continuously release zinc ions over a period of 70 h. The pore and chemical structure were, respectively, performed using scanning electron microscopy (SEM) and Fourier transform infrared spectroscopy (FT-IR). And its comprehensive properties, such as gel content, water evaporation ratio and swelling behavior were investigated. The hydrogels showed potential application value in the hydrogel dressing for zinc ion delivery.

## 1. Introduction

Zinc-containing medical dressings could locally supplement zinc at the wound site. They could effectively promote the healing of burns [1,2,3], traumatic injuries [4], surgical wounds, ulcers [5] and skin inflammation [6]. These dressings also stimulated epidermal cell proliferation and accelerated re-epithelialization by modulating the skin’s inflammatory response [7]. Zinc-containing medical dressings were available in various forms, including aerogels [8], hydrogels [9,10], sprays and films [11,12,13].

Hydrogel had a three-dimensional network structure, which was similar to the extracellular matrix (ECM) [14,15,16]. Their porous nature enables sustained drug release, making them widely applicable in medical dressing applications.

Polyvinyl alcohol (PVA) can form a highly water content network through cross-linking, effectively mimicking the extracellular matrix environment. The high water content of PVA helps maintain a moist wound environment. It can accelerate autolytic debridement of the wound [17]. Chitosan (CS) is a natural polysaccharide [18]. It exhibited excellent biocompatibility and low cytotoxicity [19,20,21]. The positively charged amino structure on the CS main chain could engender electrostatic interactions with anionic groups on the microorganism’s cell membrane [22]. This provides it with antibacterial properties. Its hydrophilic groups can effectively retain moisture [23].

Hydrogels can be prepared by physical cross-linking and chemical cross-linking [24]. Physical cross-linking is achieved via non-covalent interactions such as ionic bonds, hydrophobic interactions, and hydrogen bonding [25,26]. This method does not require chemical cross-linking agents to avoid the residue of toxic substances. It offers advantages including low cost, minimal cytotoxicity, and operational simplicity [27]. Physically cross-linked hydrogels typically exhibit superior biocompatibility and degradability [28]. Chemical cross-linking relies on covalent bond formation, which commonly includes step reaction polymerization and radical polymerization [29], which encompass processes such as Schiff base reactions [30], enzyme-induced cross-linking, photo-initiated cross-linking [31] and radiation-induced cross-linking [32]. Chemical cross-linking significantly enhances the mechanical strength and structural stability of hydrogels.

In this paper, a hydrogel was prepared via gamma ray irradiation cross-linking with ^60^Co, aiming to achieve stable performance without chemical reagents. PVA solution was selected as the matrix material due to its biocompatibility, and combined with CS and zinc gluconate. The structures and properties of the hydrogels were characterized by SEM, FT-IR, gel content, water evaporation rate and swelling rate. The zinc ion release property was investigated in vitro. The antibacterial property was measured against *Staphylococcus aureus* and *Escherichia coli*. The diameter of the antibacterial zones was measured by the Digimizer digitizer software.

## 2. Materials and Methods

### 2.1. Materials

Zinc gluconate (purity 98%) was purchased from Shanghai Aladdin Biochemical Technology Co., Ltd. (Shanghai, China) CS (90–91% deacetylated, viscosity is 85 mPa·S) was obtained from Zhejiang Golden-Shell Biological Co., Ltd. (Yuhuan, China) PVA (polymerization degree 1700, alcoholics degree 87–89%) was purchased from Aladdin Industrial, Inc. (Shanghai, China). *Staphylococcus aureus* (CMCC 26003) and *Escherichia coli* (ATCC 8739) were purchased from Guangdong Provincial Microbial Bacteria Preservation Center (Guangzhou, China). Glacial acetic acid was purchased from Tianjin Zhiyuan Reagent Co., Ltd., (Tianjin, China) and the phosphate-buffered saline (PBS) solution was supplied from Shanghai Yuanmu Biotechnology Co., Ltd. (Shanghai, China) Fluorescent reagents (Calcein AM, G1728-0.1 ML, Servicebio, Wuhan, China).

### 2.2. Preparation of CS/PVA/Zinc Gluconate Hydrogel

PVA powder (5 g) was added to distilled water and stirred continuously for 1 h at 90 °C to prepare a 5 wt % PVA solution. Zinc gluconate powder (1 g) and CS powder (0, 0.5, 1, 1.5, 2, 2.5 and 3 g) were added to the PVA solution to prepare homogeneous suspensions. Subsequently, acetic acid, amounting to one-third of the mass of CS, was added to the suspension to prepare the CS/PVA/zinc gluconate solution. The pH value of the final mixtures was 6. The samples were designated as CS-0, CS-0.5, CS-1, CS-1.5, CS-2, CS-2.5 and CS-3, respectively, as presented in Table 1.

PVA powder (5 g) was added to distilled water and stirred continuously for 1 h at 90 °C to prepare a 5 wt % PVA solution. CS powder (1 g) and zinc gluconate powder (0, 0.5, 1, 1.5, 2, 2.5 and 3 g) were added to the PVA solution to prepare homogeneous suspensions. Subsequently, acetic acid, amounting to one-third of the mass of CS, was added to the suspension to prepare the CS/PVA/zinc gluconate solution. The pH value of the final mixtures was 6. The samples were designated as Zn-0, Zn-0.5, Zn-1, Zn-1.5, Zn-2, Zn-2.5 and Zn-3, respectively, as presented in Table 2.

**Figure 1:**
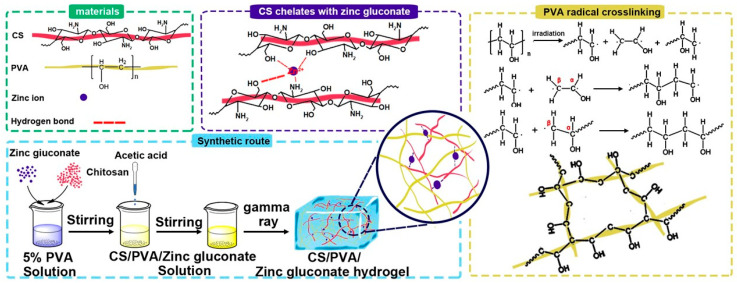
The preparation of CS/PVA/zinc gluconate hydrogels involved possible reactions.

The CS/PVA/zinc gluconate solutions were placed in a room-temperature vacuum environment at −0.1 MPa for 12 h to eliminate bubbles. Then the solutions were transferred into centrifuge tubes and purged with nitrogen gas for 5 min to achieve an oxygen-free environment. The solutions underwent gamma-ray irradiation (60 kGy) using a ^60^Co facility to prepare CS/PVA/zinc gluconate hydrogels at room temperature with a dose ratio at 15–20 Gy/min. Figure 1 shows the preparation of CS/PVA/zinc gluconate hydrogels involved in possible reactions.

After irradiation, the C-C single bonds on the PVA macromolecular chains were destroyed, forming a large number of double free radicals. The free radicals freely combine with each other or form associations with free O-H, forming a complex cross-linked network structure [33]. 

### 2.3. Scanning Electron Microscopy Analysis

The hydrogel samples were frozen for 12 h, and placed in a freeze dryer for 48 h to obtain freeze-dried hydrogel samples. Scanning electron microscopy (SEM, JSM-7500F, Joel, Portland, OR, USA) was used to observe the morphology of freeze-dried hydrogel. The pore size and porosity of the hydrogel were measured by ImageJ (https://ruanjian.bzyinxiao6.com/soft/133478.html?qhclickid=037d6af1c140e7e5, accessed on 7 November 2025).

### 2.4. Fourier Transform Infrared (FT-IR) Spectroscopy

The hydrogel samples were frozen for 12 h, and placed in a freeze dryer for 48 h to obtain freeze-dried hydrogel samples. The chemical structure of freeze-drying hydrogel was characterized by FT-IR (Tensor II FTIR, Bruker, Billerica, MA, USA). The spectral range was 400–4000 cm^−1^ with a resolution of 4 cm^−1^.

### 2.5. Gel Content

The hydrogels were vacuum freeze-dried for 48 h to obtain the freeze-dried hydrogel. The lyophilized hydrogels were immersed in distilled water to remove the soluble part from 24 h, and then vacuumed dry at 50 °C for 48 h until they reached constant weight. Take the average of the three measurements as the result of the gel content test.

(1) $$\mathrm{Gel}\;\mathrm{content}=\frac{W_{e}}{W_{d}}\times 100\%$$

where $W_{d}$ is the quality of freeze-dried hydrogel, and $W_{e}$ is the dried gel weight after extraction in distilled water.

### 2.6. Water Evaporation Ratio

The hydrogel was immersed in distilled water until the swelling equilibrium was reached. The hydrogels were placed in an oven (DZ-2BCII, TEST, Tianjin, China) with a temperature of 50 °C. Weight of the hydrogel was measured at regular intervals until completely dry. The average of three measurements was taken as the result for the water evaporation ratio.

(2) $$\mathrm{Water}\;\mathrm{evaporation}\;\mathrm{ratio}=\frac{W_{1}-W_{2}}{W_{1}-W_{3}}\times 100\%$$

where $W_{1}$ is the initial quality, $W_{2}$ is the measured mass and $W_{3}$ is the final weight of the hydrogel.

### 2.7. Swelling Ratio

The freeze-dried hydrogel with the soluble parts removed was immersed in distilled water for 48 h (room temperature) until the hydrogel reached swelling equilibrium. The excess solvent on the surface of the hydrogel was removed by blotting quickly with absorbent paper and weighed. Swelling ratio was calculated as follows:

(3) $$\mathrm{Swelling}\;\mathrm{ratio}\;=\frac{W_{s}-W_{d}}{W_{d}}\times 100\%$$

where $W_{s}$ is the final hydrogel mass and $W_{d}$ is the freeze-dried hydrogel with the soluble parts removed mass. The average of three measurements was taken as the result for the swelling ratio.

### 2.8. In Vitro Zinc Ions Release

The in vitro release characteristics of zinc ions prepared by 1.3 g hydrogel and PBS buffer were measured (37 °C, pH 7.4). A 2 mL solution of PBS buffer and hydrogel was extracted at designated time intervals (3, 6, 9, 12, 24, 48 and 72 h); an equal amount of the fresh buffer solution was supplemented to assure the amount of PBS buffer was constant during the release process. The zinc ions in the hydrogel were diluted after being released into the aqueous solution and the release of zinc ions was measured by atomic absorption spectrometry (AAS, NovAA400P, Analytic Jena, Jena, Germany).

### 2.9. Antibacterial Properties

The antibacterial activity of the hydrogel against *Staphylococcus aureus* and *Escherichia coli* was measured using the bacteriostasis circle test. Quadrate hydrogels (1 cm diameter) were sterilized by UV irradiation for 15 min. Samples were placed on agar culture medium inoculated with bacteria. The medium was subsequently cultured at 37 °C for 24 h. The antibacterial activity of the hydrogels was reflected by the size of the inhibition zone surrounding the hydrogel in the medium, with the diameter of the inhibition zone measured using Digimizer (6.3.0) Digitizer software.

### 2.10. Cytotoxicity Testing

A mouse primary fibroblasts suspension of 100 μL was placed in the wells of a 96-well plate. The plates were precultured in the incubator for 30–45 min (37 °C, 5% CO_2_). After UV light disinfection, the samples were placed in the culture plate. After incubation, the fresh culture medium was replaced, preheated to 37 °C and incubated in the dark for 30 min at 37 °C to ensure that the cell lactonase fully hydrolyzed Calcein AM and generated Calcein with green fluorescence. The culture medium was removed, washed 2–3 times with PBS, and then added to a serum-free cell culture medium for observation under a fluorescence microscope or detection using a fluorescence enzyme-linked immunosorbent assay (ELISA) reader. The solution was measured at the absorption peak of 450 nm with a microplate reader. The mean and SD were used as the experimental results. The relative activity of the cells in the experimental and blank groups was compared. Cell viability was calculated as follows:

(4) $$\mathrm{Cell}\;\mathrm{viability}\;=\frac{{OD}_{ex}-{OD}_{b}}{{OD}_{n}-{OD}_{b}}\times 100\%$$

where ${OD}_{ex}$ is the optical density of the experimental group, ${OD}_{b}$ is the optical density of the blank background group and ${OD}_{n}$ is the optical density of blank control group. The average of the three measurements was taken as the result for cell viability.

## 3. Results and Discussion

### 3.1. Morphology of Hydrogel

**Figure 2:**
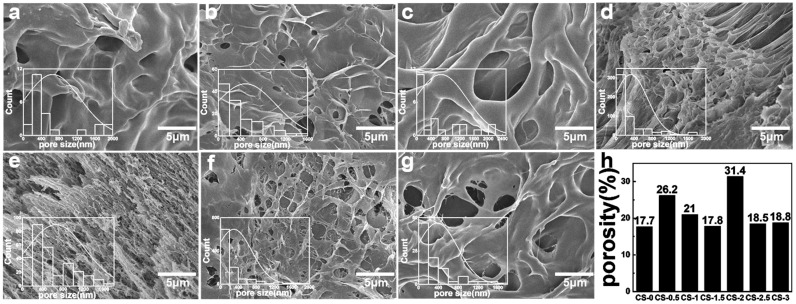
SEM of hydrogels with different CS contents: (**a**) CS-0; (**b**) CS-0.5; (**c**) CS-1; (**d**) CS-1.5; (**e**) CS-2; (**f**) CS-2.5; (**g**) CS-3; (**h**) porosity of hydrogel.

**Figure 3:**
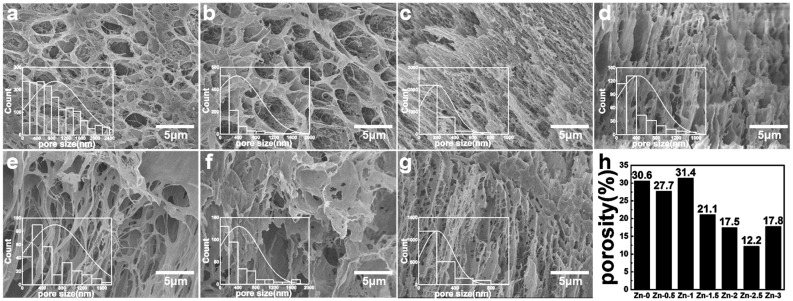
SEM of hydrogels with different zinc gluconate contents: (**a**) Zn-0; (**b**) Zn-0.5; (**c**) Zn-1; (**d**) Zn-1.5; (**e**) Zn-2; (**f**) Zn-2.5; (**g**) Zn-3; (**h**) porosity of hydrogel.

The connectivity of the hydrogel pore played a crucial role in the zinc ions’ release process. The interconnected pore structure contributed to the diffusion of zinc ions. The morphology and size of freeze-dried hydrogels were characterized by SEM. The pore size and porosity were quantitatively analyzed from SEM images using ImageJ·software. As shown in Figure 2 and Figure 3, the freeze-dried hydrogels exhibited a porous three-dimensional network structure with interconnecting pores. These pores enabled the transmission of water and wound exudates as channels for drug loading.

In Figure 2, with the increase in CS content, the pore size firstly decreased and then increased. The addition of CS affected the cross-linking of PVA, and the pore distribution of the hydrogel gradually became dense and uniform. But with the addition of CS, the pore size gradually increased and the distribution of pores became loose. The sample CS-2 exhibited a porosity of 31.35%.

In Figure 3, with the increase in Zn content, the pore size firstly decreases, secondly increases and then decreases. The pore distribution of the hydrogels was dense and uniform. The sample Zn-1 exhibited a porosity of 31.35%.

### 3.2. FT-IR Spectroscopy

**Figure 4:**
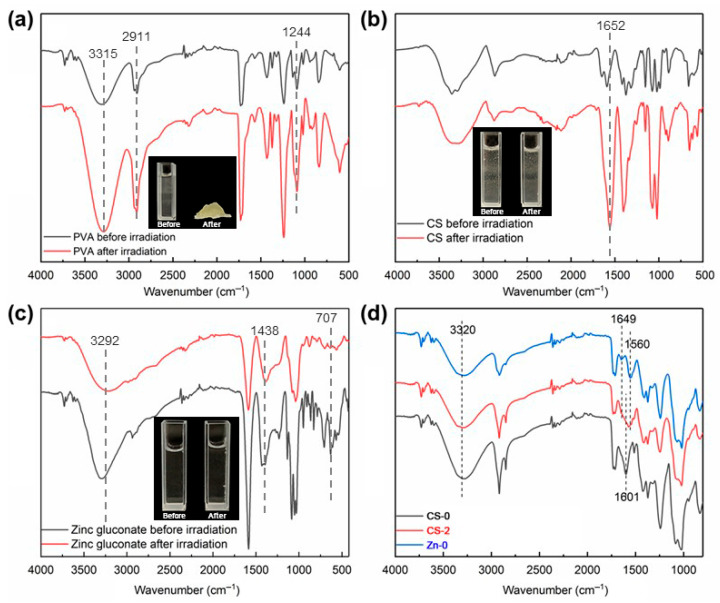
FT-IR spectra of CS, PVA, zinc gluconate and prepared hydrogel with different components. (**a**) PVA before and after irradiation; (**b**) CS before and after irradiation; (**c**) zinc gluconate before and after irradiation; (**d**) hydrogels of different components.

The samples of PVA, CS, zinc gluconate aqueous solution and the CS/PVA/zinc gluconate aqueous solution before and after irradiation were analyzed by FT-IR, and the results are presented in Figure 4.

The FT-IR results of the PVA aqueous solution sample before and after irradiation cross-linking are presented in Figure 4a. The PVA aqueous solution formed a hydrogel upon irradiation, and the FT-IR spectrum of the PVA hydrogel sample was consistent with the PVA aqueous solution sample. This showed that there was no formation of new functional groups in PVA hydrogel. The break and reconnection of C–C single bond on the main chain of PVA formed hydrogel. A strong and broad absorption band near 3315 cm^−1^ corresponded to the O–H stretching vibration, which might also contribute to the features observed at 2911 cm^−1^. The peaks at 1718 cm^−1^ and 1732 cm^−1^ were attributed to the C=O stretching vibrations of residual ester groups [34]. The signal at 1244 cm^−1^ was assigned to the bending vibration of the C–H bond in PVA, while the absorption at 1426 cm^−1^ corresponded to the CH_2_ scissoring vibration.

Figure 4b presents the FT-IR spectra of CS aqueous solution before and after irradiation. The physical picture indicated that the solution remained in a liquid state both before and after irradiation. This suggested that CS would not form a gel after irradiation. The absorption peaks near 3360 cm^−1^ were attributed to O–H stretching vibrations. The peak around 3288 cm^−1^ was correspond to N–H stretching vibrations [35]. The two bands were significantly influenced by hydrogen bonding. The peak at 2878–2860 cm^−1^ was caused by C–H stretching vibrations. The peak at approximately 1652 cm^−1^ corresponded to the amide I band (C=O). The peak at 1593 cm^−1^ corresponded to the amide II band (N–H), and the peak at 1310 cm^−1^ corresponded to the amide III band (C–N) [36]. The absorption peak at 1020 cm^−1^ was associated with C–O stretching vibrations, and the peak near 890 cm^−1^ arise from ring stretching vibrations. After irradiation, the characteristic absorption peak of the β(1→4) glycosidic bond in CS at 890 cm^−1^ shifted to 893 cm^−1^. It exhibited a decrease in vibration intensity, indicating radiation-induced scission of the glycosidic linkage. The increase in peak at 1024 cm^−1^ suggested the scission of C–O–C bonds. These results are uniformly explained by the fact that degradation reactions predominantly occurred following irradiation of CS.

Figure 4c presents the FT-IR spectra of zinc gluconate aqueous solution before and after irradiation. The strong and wide absorption peak near 3292 cm^−1^ was caused by O–H stretching vibration. The absorption peak at 2940 cm^−1^ was caused by C–H stretching vibrations. The absorption peaks near 1584 cm^−1^, 1438 cm^−1^ and 1396 cm^−1^ were caused by COO– stretching vibration [37]. The absorption peaks near 1135 cm^−1^, 1088 cm^−1^, 1056 cm^−1^ and 1034 cm^−1^ were caused by C–OH stretching vibration. No peaks appeared or disappeared after irradiating the zinc gluconate aqueous solution. This indicated that the irradiation did not change the structure of zinc gluconate aqueous solution.

The FT-IR of the CS/PVA hydrogel, PVA/zinc gluconate hydrogel and CS/PVA/zinc gluconate hydrogel are shown in Figure 4d. After the addition of CS, the characteristic peaks of the hydrogel at 3320 cm^−1^ were broadened by corresponding to O–H, free O–H and N–H, which could form intermolecular or intramolecular hydrogen bonds. This indicated that the formation of molecular associated between CS and PVA through hydrogen bonding. The peak of the COO– at 1601 cm^−1^ has shifted due to the bending vibration of the N–H on CS. There were no condensation or copolymerization reactions between CS and PVA. After the addition of zinc gluconate, the characteristic peak of –NHCO– of CS at 1649 cm^−1^ disappeared.

### 3.3. Gel Content

**Figure 5:**
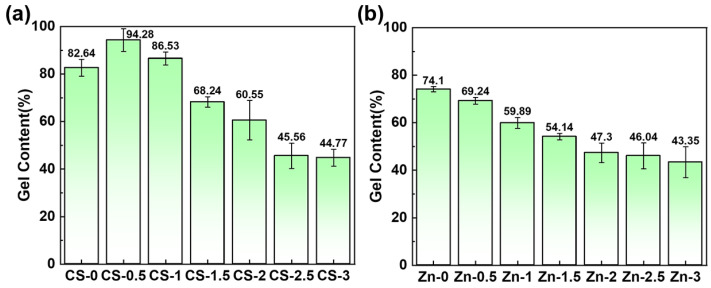
Gel content of CS/PVA/zinc gluconate hydrogel. (**a**) Effect of CS on gel content; (**b**) effect of zinc gluconate on gel content.

The gel content of the hydrogel was measured by evaluating the degree of cross-linking. The extent of cross-linking was decided by the gel content of the hydrogel. In Figure 5a, the cross-linking degree of the PVA/zinc gluconate hydrogel reaches 82.64%, which is close to the theoretical value of the cross-linking degree of the pure PVA hydrogel. The majority of PVA participated in the cross-linking reaction. When a small amount of CS was added into the PVA/zinc gluconate solution, the cross-linking degree increased to 94.28%. The CS could be wrapped in the PVA system and has not been dissolved out to distilled water. As the CS concentration increased further, the cross-linking degree gradually decreased. A large amount of CS interfered with the PVA cross-linking network, thereby reducing the overall gel content. In Figure 5b, the gel content decreases progressively with increasing zinc gluconate. The inclusion of zinc gluconate improved the overall quality, thereby reducing the proportion of the gel portion in the hydrogel. As a soluble component, zinc gluconate existed in ionic form within the gel network and could be readily removed by washing with distilled water.

### 3.4. Water Evaporation Ratio

**Figure 6:**
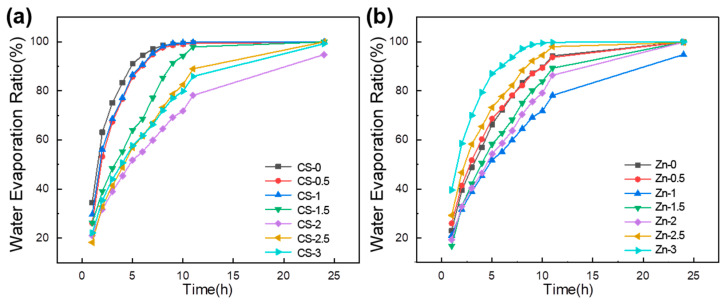
Effect of CS and Zinc gluconate on water evaporation ratio of hydrogels. (**a**) Effect of CS on water evaporation ratio; (**b**) effect of zinc gluconate on water evaporation ratio.

In Figure 6a,b, with the content of CS increasing, the water evaporation ratio of the hydrogel firstly decreases and then increases. The water evaporation ratio of the sample CS-2 was the smallest. The ratio of water evaporation was related to the porosity of the hydrogel (Figure 2). In addition, the water evaporation rate of the hydrogel was also related to the cross-linking degree of the system. With the increase in CS, the cross-linking degree of the system decreased (Figure 5), and the water evaporation rate increased.

With the content of zinc gluconate increasing, the water evaporation ratio of the hydrogel firstly decreased and then increased. A small amount of gluconic zinc was added, and zinc ions formed chelation interactions with CS. This contributed to reducing the water evaporation rate of the hydrogel. When the content of gluconic zinc increased, the zinc ions reduced the cross-linking degree of the system. This resulted in the water evaporation rate of the hydrogel increasing.

### 3.5. Swelling Ratio

**Figure 7:**
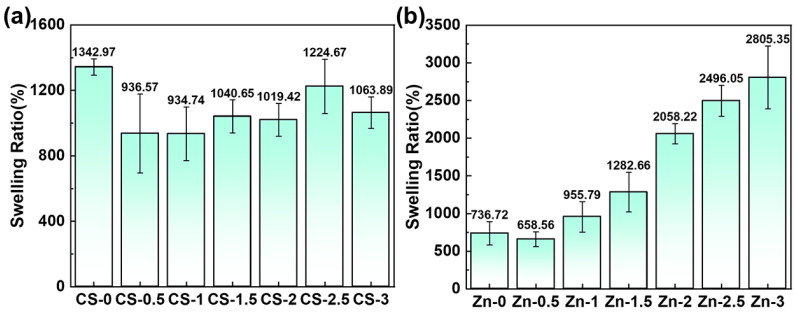
Effect of CS and zinc gluconate on swelling ratio of hydrogel. (**a**) Effect of CS on swelling ratio; (**b**) effect of zinc gluconate on swelling ratio.

The ideal wound dressing should possess the capacity to absorb exudate from the wound site, with an optimal swelling ratio ranged between 100% and 900% [37]. The influence of CS content and zinc gluconate concentration on the swelling ratio of hydrogels is shown in Figure 7a,b. The swelling ratio of the hydrogel was significantly affected by variations in CS and zinc gluconate content.

PVA molecules formed a cross-linked network The water molecules relied on the interaction with the polar groups of the molecular chains to enter the network. The content of polar groups and the closeness of the cross-linking network in the system determined the swelling ratio of the hydrogel. The swelling ratio of the PVA/zinc gluconate hydrogel reached a high value of 1342.97%. Upon addition of a small amount of CS, the CS molecule was encapsulated by the PVA molecule. It led to enhanced cross-linking density and a reduced swelling ratio, which was reduced to 936.57%. With the increase in CS concentration, the content of polar groups increased, the cross-linking degree of the hydrogel decreased (Figure 5a), the closeness of the cross-linking network decreased. This raised the swelling ratio to 1224.67%. This led to the swelling ratio of the hydrogel firstly decreasing and then increasing.

In Figure 7b, the swelling ratio increases with rising zinc ion concentration. The hydrogel without zinc ions exhibited a high cross-linking density and a correspondingly low swelling ratio of 736.72%. With increasing zinc gluconate concentration, the cross-linking density decreased (Figure 5b). Zinc ions dissolve in water, exposing more polar groups. This also created a concentration difference on both sides of the hydrogel, which could allow more water molecules to enter. It improved the swelling rate of the hydrogels. The maximum swelling ratio reached 2805.35%, an increase of 281.35%.

### 3.6. Release Properties of Zinc Ions

**Figure 8:**
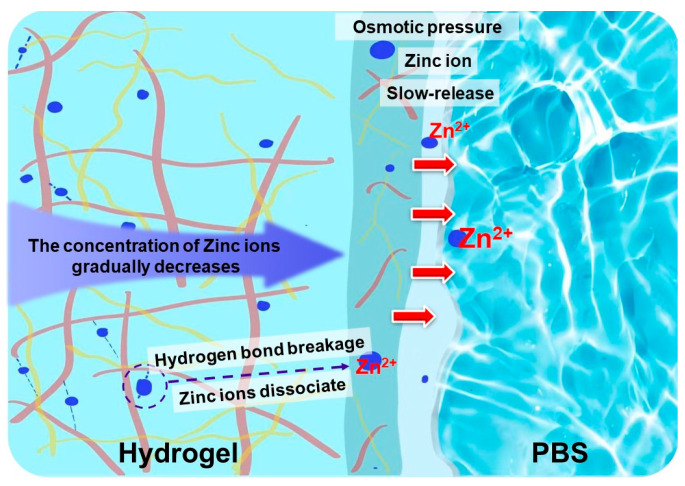
Diagram of zinc ion release process from hydrogels.

**Figure 9:**
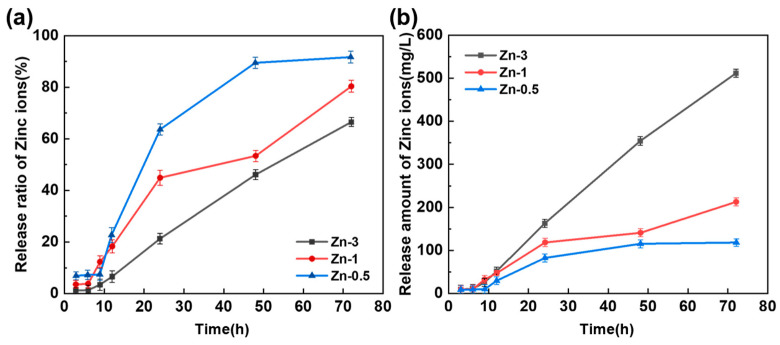
In vitro release curves of zinc ions. (**a**) Release ratio of Zinc ions; (**b**) Release amount of Zinc ions.

The release of zinc ions should be sustained within the specified time. Both a quick release rate and a high release volume could affect the wound healing. The release of Zn ions was related to the content of CS in the hydrogel system and the closeness of the cross-linking network. A schematic diagram of zinc ion release process from hydrogels to PBS is displayed in Figure 8. Zinc ions located on the hydrogel side adjacent to the PBS were preferentially released into the water. Zinc ions situated on the side distant from the PBS remaining bound to CS molecules. This enabled it to maintain original concentration in that region. This established a concentration gradient of zinc ions within the hydrogel. To maintain system equilibrium, zinc ions gradually dissociated from chelation interactions and diffused toward the region of PBS. This enabled the hydrogel to continuously release zinc ions. The content of CS in the hydrogel with different zinc ion content was fixed, the fixed zinc ion content was certain. A higher content of zinc ions in the hydrogel resulted in a greater release of zinc ions. The zinc ion released curves of CS/PVA/zinc gluconate hydrogels with different content of zinc gluconate (0.5 g, 1 g, and 3 g) are shown in Figure 9.

In Figure 9a, with the increment in the zinc ion concentration, the rate of release of zinc progressively decreases. In sample Zn-0.5, the release rate of zinc ions reached 100% at 50 h. In sample Zn-1 and Zn-3, the release rate of zinc ions was still less than 80% at 70 h. The release rate of zinc ions initially showed a slow increasing trend. As the concentration of zinc ions increases, the rate of zinc ion release decreases. This indicated that the hydrogel with a low zinc ion concentration could not release zinc ions for a long time. This might not achieve the desired therapeutic effect.

In Figure 9b, with the increment in zinc ion concentration, the quantity of zinc ions released from the hydrogel progressively increases. In sample Zn-3, the release rate of zinc ions from the hydrogel reached 600 mg/L. In samples Zn-1 and Zn-0.5, the release rates of zinc ions from the hydrogel were both under 200 mg/L at 70 h. This indicated that the hydrogel with high zinc ion concentration could continuously release a large amount of zinc ions. This could have an impact on health.

### 3.7. Antibacterial Activity Test

**Figure 10:**
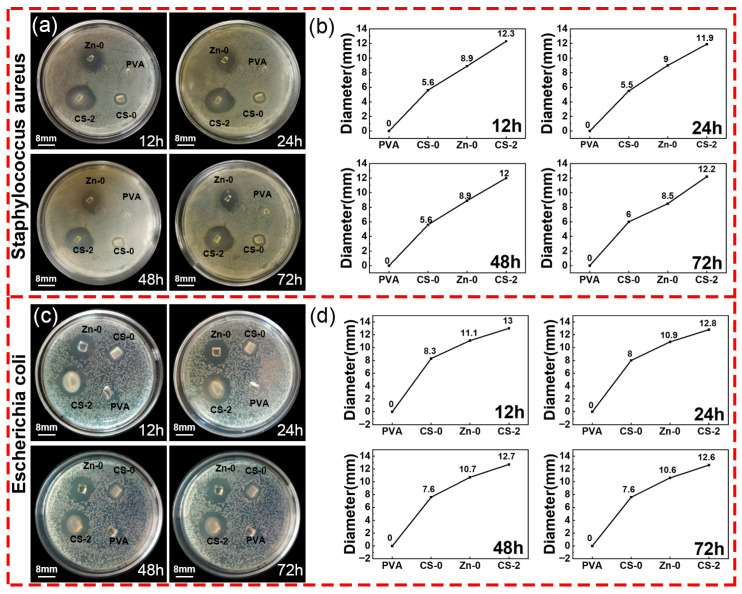
Antibacterial activities of hydrogels. (**a**,**b**) Photographs of the bacteriostatic effect on *Staphylococcus aureus* and the diameter of the inhibition zone; (**c**,**d**) photographs of the bacteriostatic effect on *Escherichia coli* and the diameter of the inhibition zone.

The ideal wound dressing should possess excellent antibacterial properties to prevent wound infection. The antibacterial properties of the CS/PVA/zinc gluconate hydrogels were evaluated with *Staphylococcus aureus* and *Escherichia coli*. In Figure 10, both CS and zinc gluconate exhibit antibacterial activity. The synergistic interaction between CS and zinc gluconate significantly enhanced the antibacterial performance of hydrogels.

In Figure 10, pure PVA hydrogel does not show antibacterial properties. The samples CS-0 and Zn-0 indicated that the PVA hydrogels containing CS or zinc gluconate possessed antibacterial properties. The sample CS-2 indicated that the synergistic effect of CS with zinc gluconate enhanced the antibacterial activity of the hydrogel. The inhibition zone diameter of the CS/PVA/zinc gluconate hydrogel was approximately 1.5 times larger than the CS/PVA hydrogel. The inhibition zone diameter of the CS/PVA/zinc gluconate hydrogel was approximately 2.2 times larger than the PVA/zinc gluconate hydrogel. This indicated that the synergistic effect of CS and zinc gluconate could significantly enhance the antibacterial performance of the hydrogel.

### 3.8. Cytotoxicity Testing

**Figure 11:**
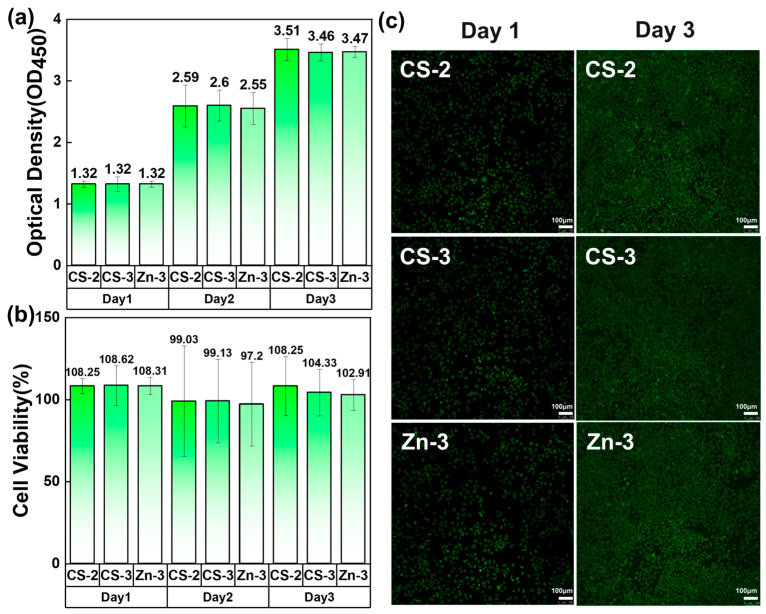
Cytotoxicity testing of hydrogels. (**a**) Optical Density of hydrogels; (**b**) cell viability of hydrogels; (**c**) live assay fluorescence images for cells.

The cytotoxicity of the nanofiber membranes was characterized using a CCK8 assay. Figure 11 shows the cell viability on different cell cultures with the addition of CS-2, CS-3, and Zn-3. After three days, the cell viability of CS-2, CS-3, and Zn-3 were 108.25%, 104.33%, and 102.91%, respectively. Figure 11a,c show that the cells in samples CS-2, CS-3, and Zn-3 could all survive well. The survival rates of cells with high CS and zinc ion content (CS-3 and Zn-3) were lower than that of cells with a CS/PVA/zinc gluconate ratio of 2:5:1 (CS-2). Figure 11b showed that the number of cells with added CS-2 remained unchanged on day 3. And the cell viability of cells with CS-3 and Zn-3 added decreased by 3.95% and 4.99%, respectively. This indicates that both high concentrations of zinc ions and CS can have an impact on cell survival.

## 4. Conclusions

CS/PVA/zinc gluconate hydrogels were prepared via gamma ray irradiation. When the mass ratio of CS/PVA/zinc gluconate was 2:5:1, the hydrogel exhibited optimal comprehensive performance. The hydrogel enabled sustained release of zinc ions. Increasing the zinc gluconate content extended the zinc ion release duration to over 70 h. A synergistic effect between CS and zinc gluconate contributed to enhanced antibacterial activity. The inhibition zone of the CS/PVA/zinc gluconate hydrogel was 1.5 times larger than that of the CS/PVA hydrogel and 2.2 times larger than that of the PVA/zinc gluconate hydrogel. This ratio of hydrogel has lower cytotoxicity, and the cell viability can reach 108.25%. The incorporation of CS and zinc gluconate improved the porosity and pore size of the hydrogel. This was conducive to the transfer of ions. Water evaporation and swelling tests indicated that these hydrogels possessed excellent water retention capacity and high-water absorption. It could provide an environment to promote wound healing. These results suggested that CS/PVA/zinc gluconate hydrogels had excellent zinc ion release, low toxicity and antibacterial performance, and held promising potential for clinical application as wound dressings.

## Figures and Tables

**Table 1:** Mixed solution of CS/PVA/zinc gluconate with different CS content.

Name	PVA	CS-0	CS-0.5	CS-1	CS-1.5	CS-2	CS-2.5	CS-3
CS–PVA–Zinc gluconate	0:5:0	0:5:1	0.5:5:1	1:5:1	1.5:5:1	2:5:1	2.5:5:1	3:5:1

**Table 2:** Mixed solution of CS/PVA/zinc gluconate with different zinc gluconate content.

Name	PVA	Zn-0	Zn-0.5	Zn-1	Zn-1.5	Zn-2	Zn-2.5	Zn-3
CS–PVA–Zinc gluconate	0:5:0	2:5:0	2:5:0.5	2:5:1	2:5:1.5	2:5:2	2:5:2.5	2:5:3

## Data Availability

The original contributions presented in this study are included in the article. Further inquiries can be directed to the corresponding authors.
